# Multiply to conquer: Copy number variations at *Ppd-B1* and *Vrn-A1* facilitate global adaptation in wheat

**DOI:** 10.1186/s12863-015-0258-0

**Published:** 2015-07-29

**Authors:** Tobias Würschum, Philipp H. G. Boeven, Simon M. Langer, C. Friedrich H. Longin, Willmar L. Leiser

**Affiliations:** State Plant Breeding Institute, University of Hohenheim, 70593 Stuttgart, Germany; Current address: Bayer CropScience Aktiengesellschaft, European Wheat Breeding Center, 06466 Gatersleben, Germany

**Keywords:** Wheat, Copy number variation, Ppd-B1, Vrn-A1, Adaptation, Flowering time

## Abstract

**Background:**

Copy number variation was found to be a frequent type of DNA polymorphism in the human genome often associated with diseases but its importance in crops and the effects on agronomic traits are still largely unknown.

**Results:**

Here, we employed a large worldwide panel of 1110 winter wheat varieties to assess the frequency and the geographic distribution of copy number variants at the *Photoperiod-B1* (*Ppd-B1*) and the *Vernalization-A1* (*Vrn-A1*) loci as well as their effects on flowering time under field conditions. We identified a novel four copy variant of *Vrn-A1* and based on the phylogenetic relationships among the lines show that the higher copy variants at both loci are likely to have arisen independently multiple times. In addition, we found that the frequency of the different copy number variants at both loci reflects the environmental conditions in the varieties’ region of origin and based on multi-location field trials show that *Ppd-B1* copy number has a substantial effect on the fine-tuning of flowering time.

**Conclusions:**

In conclusion, our results show the importance of copy number variation at *Ppd-B1* and *Vrn-A1* for the global adaptation of wheat making it a key factor for wheat success in a broad range of environments and in a wider context substantiate the significant role of copy number variation in crops.

**Electronic supplementary material:**

The online version of this article (doi:10.1186/s12863-015-0258-0) contains supplementary material, which is available to authorized users.

## Background

The plethora of QTL mapping studies performed during the last decades has shown that the genotypic variation of agronomically important traits in crops is to a great extent controlled by polymorphisms in the nuclear DNA. Owing to the available genotyping technologies at the time, these studies were almost exclusively based on single nucleotide polymorphisms (SNPs) and small insertions-deletions (INDELs) which were consequently assumed to be the major types of DNA polymorphism underlying genotypic variation. In the last decade however, a different type of DNA polymorphism was found to be abundant in the human genome [1, 2]. Copy number variation (CNV) affects the human phenotype and was often found to be associated with diseases (e.g., [3–5]). In crops by contrast, the frequency of different copy numbers at a specific locus, their geographic distribution and their effects on the genotypic variation are still largely unknown.

Copy number variation refers to rearrangements of genomic sequences which typically are larger than 1 kb, resulting in the loss or gain of these DNA segments [6]. Notably, in polyploid plants like wheat, copy number variation refers to the number of copies per haploid genome. While most CNVs occur in intergenic regions [7], this type of structural polymorphism can also encompass protein-coding genes or sequences regulating the expression of genes. Such changes in the number of functional copies or regulatory elements can in turn result in altered expression levels of these genes. Recent genome-wide studies in Arabidopsis showed that while copy number variation is present, only few of the true CNV polymorphisms result in differentially expressed genes which led to the conclusion that CNV is likely to have only a small impact on the phenotype [8]. By contrast, work in barley revealed for example, that increased boron toxicity tolerance is due to an increased copy number of a boron transporter [9] and that CNV of *CBF* genes affects abiotic stress tolerance [10]. Likewise in soybean, copy number variation of a genomic segment encompassing three genes at the *Rhg1* locus has been shown to mediate resistance against soybean cyst nematode [11]. However, in contrast to humans, the effects of CNV on the phenotype of crops are just beginning to be understood [6].

One of the prime examples for the effect of copy number variation on an important trait in crops is flowering time in wheat (*Triticum aestivum* L.) [12]. Variations in wheat flowering time have previously been shown to be caused by mutations in the *Photoperiod-1* (*Ppd-1*) and *Vernalization-1* (*Vrn-1*) genes [13]. The *Ppd-1* genes encode members of the pseudo-response regulator (PRR) family and *Vrn-1* encodes a MADS-box transcription factor which is upregulated during the vernalization process. The *Photoperiod-1* homoeolog on the D genome (*Ppd-D1*) is the most important photoperiod regulator in wheat and the photoperiod insensitive allele is caused by a large deletion upstream of the coding region which increases expression of the gene [14–16]. By contrast, the chromosomal region containing *Ppd-B1* was identified by genetic mapping but sequencing revealed no candidate mutation in this gene [14]. However, *Ppd-B1* has recently been shown to be present in different copy numbers which alter photoperiod sensitivity [12]. Wheat genotypes with one copy of the gene are photoperiod sensitive while a higher number of copies (2–4 copies) make the plants day-neutral and thus early flowering. While this initial study was based on phenotypic data from controlled greenhouse conditions and a comparably limited set of lines, Cane et al. [17] and Langer et al. [16] investigated *Ppd-B1* CNV in a quantitative genetic context, i.e., a larger number of genotypes, and based on field trials. Both found different copy numbers to be present in Australian and European wheat, respectively, and reported an effect of *Ppd-B1* copy number on flowering time. *Vernalization-1* (*Vrn-1*) is responsible for the variation in vernalization requirement and Díaz et al. [12] showed that wheat plants with an increased copy number of *Vrn-A1* have an increased requirement for vernalization. However, the frequency of different copy numbers at these two important loci and their effects in wheat varieties from different worldwide origins are still unknown.

The aim of this study was to bridge this gap and to further increase our knowledge of copy number variation in crops. We therefore investigated the phylogenetic origin, the frequency, and the geographic distribution of copy number variants at *Ppd-B1* and *Vrn-A1* in a worldwide panel of 1110 winter wheat cultivars and in addition assessed their effects on flowering time.

## Results

**Fig. 1:**
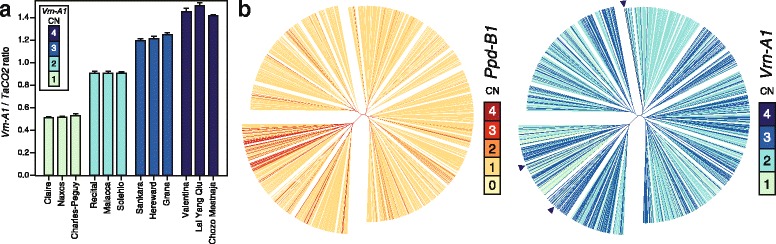
Copy number variants and their distribution in the worldwide winter wheat panel. **a** Copy number variation at the *Vernalization-A1* (*Vrn-A1*) locus was estimated from the *Vrn-A1*/*TaCO2* signal ratio. Means and standard deviations from eight measurements are shown. **b** Genetic relationships among the 1110 varieties and their copy number at the *Photoperiod-B1* (*Ppd-B1*) and *Vrn-A1* loci. The three individuals carrying the novel *Vrn-A1* four copy variant are indicated by arrowheads

This study was based on 1110 winter wheat varieties from all over the world but with a focus on European varieties (Table 1, Additional file 1: Table S1). Three copy number variants were observed for *Ppd-B1*, having 1, 2 or 3 copies (Table 1). In addition, the variety ‘Naridana’ which was registered in Poland in 2006 was found to have no copy of *Ppd-B1*. Most of the varieties studied here carried one copy (90.6 %) and only few had two (5.1 %) or three copies (4.1 %) of *Ppd-B1*. For *Vrn-A1* we observed the known alleles with 1, 2, or 3 copies but also three varieties with a copy number of four (Fig. 1a). The three varieties were ‘Valentina’ registered 1994 in Croatia, ‘Lai Yang Qiu’ from China and ‘Chozo Mestnaja’ registered 1963 in the former Soviet Union. These three varieties and three varieties for each of the 1, 2, or 3 copy number variants were again assessed for copy number variation with eight replications. This analysis revealed only a small standard deviation for the measurements of each variety but a clear difference between the four copy number variants. T-tests showed that the classes were significantly different (*P* < 0.001) thus confirming the novel copy number variant at *Vrn-A1*. For *Vrn-A1*, 7.0 % of the varieties had one copy, 48.3 % had two copies, 44.4 % had three copies, and the three varieties carrying four copies represent 0.3 %.

**Table 1:** Effect of copy number variation at *Photoperiod-B1* (*Ppd-B1*) on heading date (HD)

Genotypes	*n*	Mean HD of *Ppd-B1* copy number			*Ppd-D1*		*Ppd-B1* copy number	
		1	2	3	*p*_*G*_	α-effect	*p*_*G*_	CNV effect
All	1110	161.7 ± 4.9	157.3 ± 5.3	150.3 ± 5.7	48.2	−4.3	8.3	−3.6
EU	958	162.2 ± 4.4	159.1 ± 3.3	156.2 ± 3.0	38.7	−3.8	2.6	−2.2
SE + DK	51	168.1 ± 4.6	-	-	-	-	-	-
GB	121	163.5 ± 3.1	160.4	161.5	11.6	−3.8	1.0	−1.5
NL + BE	42	164.8 ± 3.4	161.1 ± 0.6	-	13.6	−4.1	9.3	−3.8
DE	287	162.8 ± 2.8	159.3 ± 2.5	-	6.4	−3.5	4.0	−3.6
PL	58	163.1 ± 4.8	160.1 ± 3.8	-	35.1	−3.5	2.6	−2.4
AT + CSK	61	160.2 ± 3.6	159.9 ± 3.8	157.7	23.1	−3.1	0.1	0.3
FR	232	161.4 ± 4.0	159.0 ± 3.7	157.2 ± 2.4	44.5	−3.3	7.3	−2.9
IT	25	157.4 ± 4.4	158.1	154.0 ± 2.3	38.4	−2.4	8.5	−1.4
YUG	28	154.1 ± 3.2	155.7 ± 1.6	157.8 ± 4.0	24.4	−1.7	4.5	1.2
US	37	159.7 ± 4.6	152.9	156.0 ± 0.2	-	-	8.0	−2.2
CN	46	148 ± 4.5	145.1 ± 2.4	146.4 ± 3.7	29.4	−2.9	7.9	−1.2

Mean heading date ± standard deviation, proportion of explained genotypic variance (*p*_*G*_ in %) and allele substitution (α) or copy number variation (CNV) effect in different groups of genotypes. *AT* Austria, *BE* Belgium, *CN* China, *CSK* former Czechoslovakia, *DE* Germany, *DK* Denmark, *EU* Europe, *FR* France, *GB* Great Britain, *IT* Italy, *NL* The Netherlands, *PL* Poland, *SE* Sweden, *US* United States of America, *YUG* former Yugoslavia, Serbia, Croatia

In order to evaluate the relatedness of the copy number variants at *Ppd-B1* and *Vrn-A1* and their possible origins, we employed genome-wide marker data to assess the genetic relationships among the 1110 varieties and combined the resulting neighbor-joining trees with the copy number of the individuals at the two loci (Fig. 1b, Additional file 1: Figure S1). This revealed that for *Ppd-B1* most of the three copy variants are found within the cluster containing the Chinese varieties but also in other phylogenetically distinct clusters. Similarly, the two copy variant was found in a few groups of closely related varieties but also throughout all clusters of the phylogenetic tree. Likewise for *Vrn-A1*, all copy number variants were found in the different clusters but again with a tendency of groups of closely related varieties to share the same copy number.

**Fig. 2:**
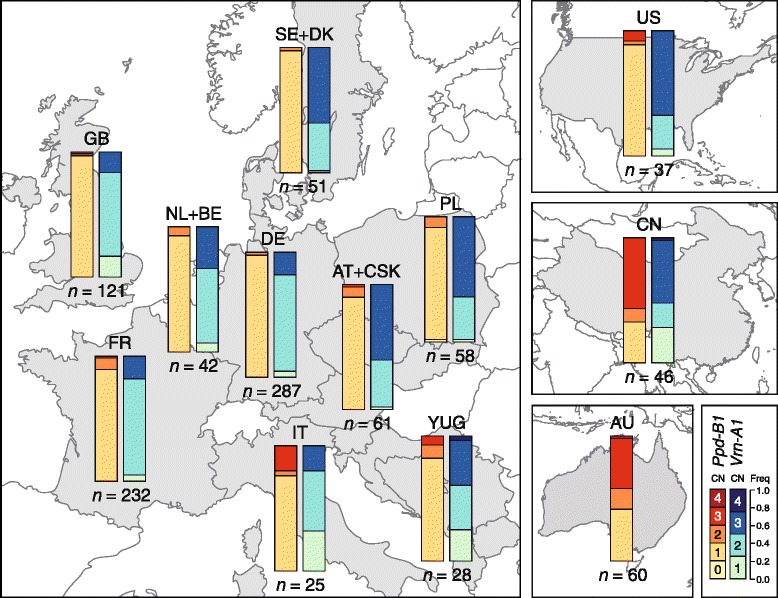
Geographic distribution of copy number variations at *Photoperiod-B1* (*Ppd-B1*) and *Vernalization-A1* (*Vrn-A1*)

We next assessed the frequency of the different copy number variants at *Ppd-B1* and *Vrn-A1* dependent on the geographic origin of the varieties, as for most of them the country of origin was known. This analysis revealed varying frequencies of both *Ppd-B1* and *Vrn-A1* in different geographic regions (Fig. 2, Additional file 1: Figure S2). Within Europe, *Ppd-B1* is mainly present as the photoperiod sensitive one copy variant but showed a clear trend from North to South. In the countries of Northern and Central Europe, only very few varieties carry a higher copy number and in the Scandinavian countries Sweden and Denmark only the one copy variant occurs. In France some more varieties with the two copy variant are found but the photoperiod insensitive two or even the three copy variant are mainly present within the Italian varieties and those from the Balkan region (the former Yugoslavia). The frequency observed for the US American varieties was similar to that found for the Southern European countries, with mainly the one copy variant but also some two or three copy variants. By contrast, more than half of the Chinese and the Australian varieties have more than one copy of *Ppd-B1* and in China the three copy variant is even the most frequent one with 56.5 %.

A similar dependency of the allele frequency on the geographic origin of the varieties was also observed for *Vrn-A1* (Fig. 2, Additional file 1: Figure S2). Within Europe, the distribution of the higher copy variants mirrors the climatic conditions present in the country of origin. The three copy variant is the major allele in the Scandinavian countries Sweden and Denmark, as well as in the countries with a more continental climate, such as Germany, Poland, Austria, and the former Czechoslovakia. By contrast, in the Netherlands, Belgium, Great Britain and in France, the two copy variant is the predominant allele. The one copy variant was also found in some varieties from Great Britain but mainly in the varieties from Southern Europe. In the US and in China the three copy variant is the prevalent allele but in China also a substantial number of varieties with the one copy variant are found.

**Fig. 3:**
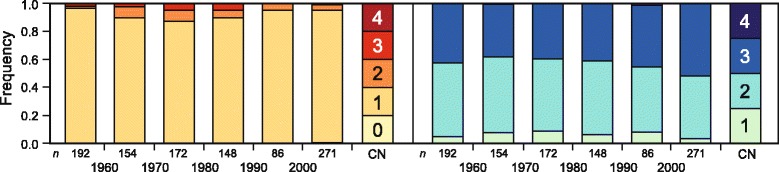
Temporal distribution of copy number variations at *Photoperiod-B1* (*Ppd-B1*) and *Vernalization-A1* (*Vrn-A1*) in 1110 winter wheat varieties from different registration periods

We next assessed the frequency of the different copy number variants over time, i.e., dependent on the year of release of the varieties (Fig. 3). This revealed that also in the earliest varieties included in this study, i.e., before 1960, all copy number variants were present and the frequency of these alleles has changed only little over time.

To evaluate the effects of the different copy number variants at *Ppd-B1* and *Vrn-A1* on heading time of wheat under field conditions, all 1110 varieties were assessed in multi-location field trials. The heritability across all test locations was 0.94 illustrating the high quality of the phenotypic data. We found that increasing *Ppd-B1* copy number generally decreased the days to heading as the average heading date was 161.7, 157.3 and 150.3 for the one, two and three copy variants, respectively (Table 1). These means were significantly different at *P* < 0.05 based on Tukey-HSD. We also genotyped the varieties for *Ppd-D1* to account for the effects of this major photoperiod regulator when estimating the effects of *Ppd-B1* copy number variation. In the complete panel of 1110 varieties, *Ppd-D1* explained 48.2 % of the genotypic variance while *Ppd-B1* accounted for 8.3 %. Within the European varieties, *Ppd-D1* explained 38.7 % of the genotypic variance and *Ppd-B1* only 2.6 %. The variance explained by *Ppd-D1* and *Ppd-B1* varied substantially between the different European countries. The explained variance of *Ppd-D1* was highest within the French and Italian varieties and for *Ppd-B1* within the varieties originating from the Netherlands and Belgium, Italy, and France. *Ppd-B1* explained only a small proportion of the genotypic variance within the varieties from Austria, and the former Czechoslovakia, as well as within those from Great Britain and Poland. By contrast, within both the US and the Chinese varieties *Ppd-B1* explained approximately 8 % of the genotypic variance. Notably, within the varieties from the Balkan region (YUG), increasing *Ppd-B1* copy number appeared to slightly increase the average time required until heading. However, the two copy number variant included only three varieties and the three copy number variant two varieties, suggesting that this may also be a sampling effect.

The analysis of the effects of *Vrn-A1* copy number on heading date in the field revealed that only within the US American varieties and within the varieties from the Netherlands and Belgium *Vrn-A1* copy number explained a substantial proportion of the genotypic variance with 22.6 and 9.0 %, respectively (Additional file 1: Table S2). However, while in the former increasing copy number delayed the time to heading, it decreased time to heading in the latter.

## Discussion

The genetic control of flowering time is an important adaptive trait for plants that affects their reproductive success and in elite breeding material is a critical component for crop yield. The diploid ancestors and the wild type bread wheat are winter annual long day plants [12] but the domestication process and selection have altered these requirements to allow the adaptation of wheat to a wide range of environmental conditions. Wheat is photoperiod sensitive and thus requires a certain day length to initiate flowering. Photoperiod insensitive mutant variants by contrast are day length neutral and flower early irrespective of the day length. This early flowering can be advantageous as it for example allows the plants to escape heat stress in regions such as Southern Europe. Díaz et al. [12] have recently shown that in addition to the known mutations in major genes caused by insertions, deletions or point mutations, copy number variation at the *Ppd-B1* and *Vrn-A1* loci affects flowering time and vernalization requirement, respectively. An increased number of *Ppd-B1* copies conferred an early flowering, day length neutral phenotype and led to an increased expression level, particularly at times when the expression of wild-type alleles is low. An increased copy number of *Vrn-1* resulted in a slower induction of expression during vernalization and consequently in an increased period of cold required to potentiate flowering [12]. As both the photoperiod and the vernalization pathway affect flowering time, we investigated the role of copy number variation at both the *Ppd-B1* and *Vrn-A1* loci in the global adaptation success of wheat.

### Copy number variants at *Ppd-B1* and *Vrn-A1*

It was previously shown that ‘Chinese Spring’ has a truncated version of *Ppd-B1* in addition to the intact copy [14]. Díaz et al. [12] further showed that in ‘Chinese Spring’ the *Ppd-B1* locus is actually comprised of three intact copies and one truncated copy, all located next to each other. Notably, the assay for *Ppd-B1* copy number variation detects both the intact and the truncated copies. In our panel of 1110 varieties, we identified four variants of *Ppd-B1* with a maximum copy number of three but not the four copy variant. This is in contrast to Cane et al. [17] who identified five *Ppd-B1* copy number variants in Southern Australian wheat, i.e., 0 to 4 copies. In contrast to Díaz et al. [12] who observed the four copy allele only in ‘Chinese Spring’, this allele was present in several modern Australian cultivars, probably due to these cultivars sharing a common ancestor which was crossed with ‘Chinese Spring’. Similar to Cane et al. [17] who observed the *Ppd-B1* null allele in two lines which share a common parent, we identified one variety with this allele. This indicates that this allele is not only rare in Australian wheat but also in wheat varieties from other major growing regions. Most of the varieties studied here carried the one copy variant and only 4-5 % the two or three copy variants which is likely also attributable to the geographic sampling of the varieties.

For *Vrn-A1*, we identified a novel copy number variant with four copies. However, as only three varieties were identified for this copy number variant, the effects of this novel variant could not be estimated and especially its effect on vernalization requires further research. In the context of the origin of *Ppd-B1* variants, Díaz et al. [12] suggested that the higher copy number variants first arose by breakage and repair resulting in the two copy variant followed by unequal crossing-over generating the three and four copy variants. The three varieties carrying this novel *Vrn-A1* copy number variant originate from Croatia, China and the former Soviet Union and thus from geographically distinct regions. In addition, they were also genetically distinct belonging to different clusters (Fig. 1b) suggesting that this copy number variant may not trace back to a common ancestor but may have arisen independently.

We observed that for both *Ppd-B1* and *Vrn-A1* all copy number variants were found in phylogenetically distinct clusters (Fig. 1b). While there is always the possibility that some varieties are misplaced in the phylogenetic tree, this occurrence of individuals with similar copy number in distinct groups suggests independent origins also for other copy number variants at *Ppd-B1* and *Vrn-A1*. Alternatively, the different copy number variants may have originated before the differentiation of winter wheat into the different clusters. However, independent origins of the copy number variants appears more likely, which consequently implies that for each copy number variant different alleles with different functionality may exist depending on the allelic version of the gene that was multiplied.

### Geographic distribution of *Ppd-B1* and *Vrn-A1* copy number variants

We observed a clear North to South trend of the frequency of different *Ppd-B1* copy number variants in Europe with the higher copy, day length neutral variants occurring mainly in Southern Europe while in Scandinavian varieties only the one copy variant occurs (Fig. 2). Conversely, for *Vrn-A1* the higher copy variants conferring increased vernalization requirement were found at higher frequencies in Northern Europe and in the countries with a more continental climate. The much higher frequency of the two and three copy variants of *Ppd-B1* in Chinese varieties as compared to the European ones is likely due to the different latitudes covered by these two regions. While both stretch over approximately 20° of latitude, the north of China corresponds to the latitude of Southern Europe. The two regions therefore strongly differ in their photoperiodic conditions with a higher pressure towards photoperiod insensitivity in Chinese varieties. The same applies to Australia where also a higher number of the more photoperiodic insensitive two and three copy variants were found. This geographic distribution underlines the important role of *Ppd-B1* and *Vrn-A1* copy number variation in the adaptation of wheat to different climates, photoperiodic conditions and vernalization requirements. This is unlikely to occur by chance and rather due to the selection of favorable phenotypes by breeders or farmers.

### Effects of copy number variation on flowering time in the field

The flowering time data obtained in the multi-location field trials confirmed *Ppd-D1* as the major regulator of flowering time under field conditions. The results further substantiated the effect of *Ppd-B1* copy number variation on flowering time in the field as higher copy numbers resulted in significantly earlier flowering (Table 1). As a general trend, we observed that within varieties from countries covering several degrees of latitude all *Ppd-B1* copy number variants were present and the locus explained a higher proportion of genotypic variance. It must be noted that the trial locations employed here were all located in Southern Germany at approximately the same latitude and consequently, the effects of *Ppd-B1* copy number variation on flowering time may be stronger when photoperiodically contrasting locations are used. Nevertheless, this shows the effect of *Ppd-B1* on the fine-tuning of flowering time in varieties from all investigated regions of origin illustrating its importance for the worldwide adaptation of wheat. The effect of *Vrn-A1* copy number variation on flowering time was negligible while its effect on vernalization requirement could not be studied in the field as the plants were all fully vernalized and consequently requires further research.

Cane et al. [17] reported that in Australian wheat the three and four copy variants of *Ppd-B1* reduced the days to heading whereas the two copy variant increased it. We observed that in contrast to the general trend of a decreased flowering time with increasing *Ppd-B1* copy number, the varieties from the Balkan region flowered later with higher copy numbers. While in this case it may well be a sampling effect, this illustrates that the copy number itself does not provide any information on the functionality of the copies. Notably, the assays employed here only assess the number of copies for these two loci but not their functionality. This means that copies may either be truncated as in ‘Chinese Spring’ or for other reasons non-functional, but also that copies can possess different functional properties. For example, Díaz et al. [12] have shown that two allelic variants of *Vrn-A1* exist of which either one was found to be duplicated in different varieties. This corroborates our finding on the multiple origins of the different copy number variants (Fig. 1B) and suggests functionally different alleles within copy number variants. Furthermore, Sun et al. [18] recently investigated the DNA methylation pattern of *Ppd-B1* and showed that this is closely correlated with copy number variation. Lines with higher copy numbers showed higher DNA methylation patterns whereas the one copy allele had lower methylation patterns. This methylation includes an important region in the 5′ upstream region which is deleted in photoperiod insensitive *Ppd-A1* and *Ppd-D1* alleles. Sun et al. [18] speculate that hypermethylation of this regulatory region may prevent the binding of repressors of *Ppd-B1* expression resulting in increased expression and consequently in photoperiod insensitivity. These additional sources of variation are not considered in the current analysis and may also affect the estimates of the different copy number variants on flowering time in the field.

## Conclusions

Our analyses based on a large worldwide panel of wheat varieties revealed that *Ppd-B1* and *Vrn-A1* copy number variants show a clear geographic pattern consistent with their roles in facilitating the worldwide adaptation of wheat. Their distribution in the phylogenetic tree suggests multiple origins of the higher copy variants and consequently alleles with different functionality within each copy number variant, adding additional complexity which requires further characterization at the molecular level. In conclusion, our results provide further support for the significant role of copy number variation in crops and in particular for the adaptation of wheat to a broad range of environments as the basis for its worldwide success.

## Methods

### Plant materials and field experiments

A panel of 1110 winter wheat (*Triticum aestivum* L.) varieties was used for this study (Additional file 2). Genotypes were released during the past decades and are from all over the world but with a focus on European varieties. The field experiments for flowering time were conducted in partially replicated designs [19] with 460 genotypes in 2012 and all 1110 genotypes in 2013 at three and four locations, respectively. The locations were Hohenheim (48° 42′ 50″ N, 9° 12′ 58″ E, 400 m above sea level (asl)), Ihinger Hof (48° 44′ 50″ N, 8° 55′ 18″ E, 493 m asl), Oberer Lindenhof (48° 28′ 26″ N, 9° 18′ 12″ E, 700 m asl) and Eckartsweier (48° 31′ 18′″ N, 7° 52′ 17″ E, 140 m asl). Phenotypic data were analyzed as described by Langer et al. [16].

### Copy number variation

Copy numbers of *Ppd-B1* and *Vrn-A1* were detected following the protocol described by Díaz et al. [12] using, however, [6FAM-BHQ1] labeled probes for *Ppd-B1* and *Vrn-A1* and [CY5-BHQ3] for *TaCO2*, and the Roche LightCycler® 480 System in combination with the Roche LightCycler® 480 Probes Master mastermix. The data for *Ppd-B1* CNV in Australian wheat were mainly taken from Cane et al. [17] for lines that were homozygous for winter alleles at the vernalization loci *Vrn-A1*, *Vrn-B1* and *Vrn-D1. Ppd-D1* was genotyped following the method described by Beales et al. [14]. The proportion of genotypic variance (*p*_*G*_) explained by *Ppd-B1* CNV or *Vrn-A1* CNV and their effect on heading date were estimated in a linear model which simultaneously accounted for the effects of the major photoperiod regulator *Ppd-D1*. The model was *y*_*i*_ ~ *Ppd-D1* + CNV, where *y*_*i*_ is the genotypic value of the *i*th variety, *Ppd-D1* the allelic status at the *Ppd-D1* locus and CNV the copy number at the *Ppd-B1* or *Vrn-A1* locus. As *Ppd-D1* was fitted first in the model, all variance attributable to this major flowering time locus should be captured by this effect thus enabling a more realistic assessment of the genotypic variance explained by the CNV.

In addition, all lines were genotyped by genotyping-by-sequencing (GBS) at Diversity Arrays Technology (Yarralumla, Australia) using the Wheat GBS 1.0 assay. After quality checks a total of 36,555 markers remained, which were used to analyze the genetic relationships among the 1110 varieties. The neighbor-joining trees were built using the *ape* package in *R* [20].

### Availability of supporting data

The data sets supporting the article are included within the article and its additional files.

## Additional files

Additional file 1: Table S1. Copy number variations at *Photoperiod-B1* (*Ppd-B1*) and *Vernalization-A1* (*Vrn-A1*) in winter wheat dependent on the country of origin. **Table S2** Effect of copy number variation *Vernalization-A1* (*Vrn-A1*) on heading date (HD). **Figure S1** Phylogenetic relationships among the 1110 varieties from different origins and their copy number at the *Photoperiod-B1* (*Ppd-B1*) and *Vernalization-A1* (*Vrn-A1*) loci. EU, Europe; CN, China; US, United States of America. **Figure S2** Frequency of copy number variations at *Photoperiod-B1* (*Ppd-B1*) and *Vernalization-A1* (*Vrn-A1*) in different geographic regions. (PDF 170 kb) Additional file 2:
**This file contains the phenotypic data and the marker data of the winter wheat genotypes used in this study.** (TXT 34 kb)
