# Supplementary material for: Multiply to conquer: Copy number variations at Ppd-B1 and Vrn-A1 facilitate global adaptation in wheat
Source: BMC Genet. 2015 Jul 29;16:96. doi: 10.1186/s12863-015-0258-0 (PMC4518651; doi:10.1186/s12863-015-0258-0)
Supplement: Additional file 1: Table S1. — Copy number variations at Photoperiod-B1 (Ppd-B1) and Vernalization-A1 (Vrn-A1) in winter wheat dependent on the country of origin. Table S2 Effect of copy number variation Vernalization-A1 (Vrn-A1) on heading date (HD). Figure S1 Phylogenetic relationships among the 1110 varieties from different origins and their copy number at the Photoperiod-B1 (Ppd-B1) and Vernalization-A1 (Vrn-A1) loci. EU, Europe; CN, China; US, United States of America. Figure S2 Frequency of copy number variations at Photoperiod-B1 (Ppd-B1) and Vernalization-A1 (Vrn-A1) in different geographic regions. (PDF 170 kb) [file 12863_2015_258_MOESM1_ESM.pdf]

Multiply to conquer: Copy number variations at *Ppd-B1* and *Vrn-A1* facilitate global adaptation in wheat

Tobias Würschum[*], Philipp H. G. Boeven, Simon M. Langer[§], C. Friedrich H. Longin, Willmar L. Leiser

State Plant Breeding Institute, University of Hohenheim, 70593 Stuttgart, Germany

[*] Corresponding author

[§] Present address: Bayer CropScience Aktiengesellschaft, European Wheat Breeding Center, 06466 Gatersleben, Germany

Supplementary Information

This file contains the Supplementary Tables and Figures.

Table S1 Copy number variations at *Photoperiod-B1* (*Ppd-B1*) and *Vernalization-A1* (*Vrn-A1*) in winter wheat dependent on the country of origin.

Country	*n*	*Ppd-B1* copy number				*Vrn-A1* copy number			
		0	1	2	3	1	2	3	4
AF	1	-	1	-	-	1	-	-	-
AU	1	-	1	-	-	-	1	-	-
AT	49	-	44	5	-	1	17	31	-
BE	17	-	17	-	-	1	14	2	-
BG	9	-	7	-	2	-	1	8	-
CA	1	-	1	-	-	-	-	1	-
CH	15	-	15	-	-	-	11	4	-
CN	46	-	15	5	26	13	9	23	1
CSK	12	-	11	-	1	-	6	6	-
DE	287	-	280	7	-	4	109	174	-
DK	12	-	12	-	-	-	7	5	-
ES	3	-	3	-	-	-	3	-	-
FI	5	-	5	-	-	-	-	5	-
FR	232	-	208	21	3	11	179	42	-
GB	121	-	119	1	1	20	81	20	-
HR	8	-	4	3	1	2	3	2	1
HU	7	-	7	-	-	-	1	6	-
IT	25	-	19	1	5	8	12	5	-
KG	3	-	3	-	-	-	1	2	-
LV	2	-	2	-	-	-	-	2	-
MX	2	-	2	-	-	1	-	1	-
NL	25	-	22	3	-	2	11	12	-
NO	1	-	1	-	-	-	-	1	-
NZ	2	-	2	-	-	-	2	-	-
PL	58	1	52	5	-	1	20	37	-
RO	5	-	4	1	-	-	-	5	-
RU	16	-	11	4	1	1	2	13	-
RS	8	-	8	-	-	1	4	3	-
SUN	8	-	8	-	-	1	1	5	1
SE	39	-	39	-	-	1	15	23	-
TR	8	-	8	-	-	-	2	16	-
UA	3	-	3	-	-	-	-	3	-
US	37	-	33	1	3	2	10	25	-
YUG	12	-	11	-	1	4	3	5	-

AF, Afghanistan; AU, Australia; AT, Austria; BE, Belgium; BG, Bulgaria; CA, Canada; CH, Switzerland; CN, China; CSK, former Czechoslovakia; DE, Germany (including 10 varieties from former Eastern Germany); DK, Denmark; ES, Spain; FI, Finland; FR, France; GB, Great Britain; HR, Croatia; HU, Hungary; IT, Italy; KG,

Kyrgyztan; LV, Latvia; MX, Mexico; NL, The Netherlands; NO, Norway; NZ, New Zealand; PL, Poland; RO, Romania; RU, Russia; RS, Serbia; SUN, former Soviet Union; SE, Sweden; TR, Turkey; UA, Ukraine; US, United States of America; YUG, former Yugoslavia. For 30 varieties no information on the origin was available.

Table S2 Effect of copy number variation *Vernalization-A1* (*Vrn-A1*) on heading date (HD).

Genotypes	*n*	Mean HD of *Vrn-A1* copy number				*Vrn-A1*	
		1	2	3	4	p_G	CNV effect
All	1110	157.7 ± 7.2	161.6 ± 4.7	160.9 ± 5.8	154.7 ± 5.1	0.2	0.4
EU	958	160.6 ± 4.8	161.9 ± 4.3	162.3 ± 4.6	154.8	0.3	0.4
SE + DK	51	160.3	168.2 ± 4.9	168.3 ± 4.3	-	1.3	0.9
GB	121	163.2 ± 2.7	163.3 ± 2.9	164.4 ± 4.3	-	1.4	0.6
NL + BE	42	169.9 ± 3.0	164.1 ± 3.4	164.1 ± 2.6	-	9.0	-1.7
DE	287	158.2 ± 4.6	162.4 ± 2.7	163.0 ± 2.8	-	2.2	0.8
PL	58	161.8	163.7 ± 5.1	162.3 ± 4.7	-	0.6	-0.7
AT + CSK	61	155.0	160.4 ± 4.2	160.1 ± 3.2	-	1.1	0.7
FR	232	160.8 ± 3.9	161.1 ± 4.0	161.5 ± 4.1	-	0.2	0.3
IT	25	155.9 ± 3.3	156.5 ± 4.7	158.7 ± 4.2	-	0.6	0.5
YUG	28	156.0 ± 3.1	153.8 ± 3.9	154.2 ± 2.4	154.8	0.8	-0.3
US	37	153.6 ± 1.0	156.9 ± 2.4	160.6 ± 4.8	-	22.6	3.6
CN	46	145.9 ± 5.5	148.2 ± 4.5	147.1 ± 2.7	149.6	1.0	0.4

Proportion of explained genotypic variance (p_G in %) and copy number variation (CNV) effect in different groups of genotypes. AT, Austria; BE, Belgium; CN, China; CSK, former Czechoslovakia; DE, Germany; DK, Denmark; EU, Europe; FR, France; GB, Great Britain; IT, Italy; NL, The Netherlands; PL, Poland; SE, Sweden; US, United States of America; YUG, former Yugoslavia, Serbia, Croatia.

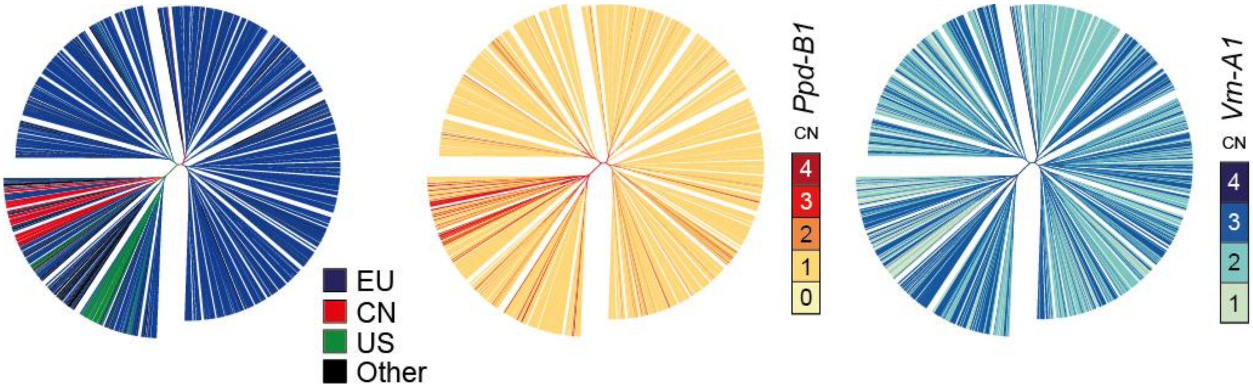

Figure S1 Phylogenetic relationships among the 1,110 varieties from different origins and their copy number at the *Photoperiod-B1* (*Ppd-B1*) and *Vernalization-A1* (*Vrn-A1*) loci. EU, Europe; CN, China; US, United States of America.

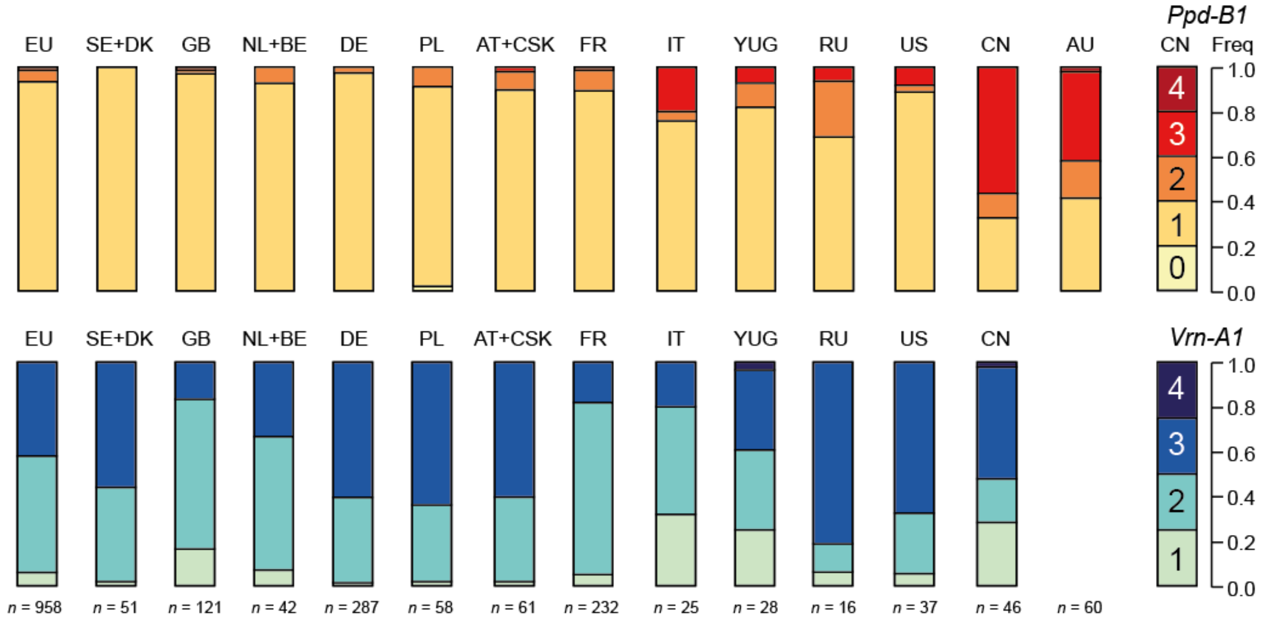

Figure S2 Frequency of copy number variations at *Photoperiod-B1* (*Ppd-B1*) and *Vernalization-A1* (*Vrn-A1*) in different geographic regions.
